# The Role of the Innate Immune System in Oncolytic Virotherapy

**DOI:** 10.1155/2017/6587258

**Published:** 2017-12-12

**Authors:** Tuan Anh Phan, Jianjun Paul Tian

**Affiliations:** Department of Mathematical Sciences, New Mexico State University, Las Cruces, NM 88001, USA

## Abstract

The complexity of the immune responses is a major challenge in current virotherapy. This study incorporates the innate immune response into our basic model for virotherapy and investigates how the innate immunity affects the outcome of virotherapy. The viral therapeutic dynamics is largely determined by the viral burst size, relative innate immune killing rate, and relative innate immunity decay rate. The innate immunity may complicate virotherapy in the way of creating more equilibria when the viral burst size is not too big, while the dynamics is similar to the system without innate immunity when the viral burst size is big.

## 1. Introduction

Oncolytic virotherapy is a promising therapeutic strategy to destroy tumors [1]. Oncolytic viruses are viruses that selectively infect and replicate in tumor cells but spare normal cells, which have two types: wild-type oncolytic viruses which preferentially infect human cancer cells, and gene-modified viruses engineered to achieve selective oncolysis. In oncolytic viral therapy, oncolytic viruses infect tumor cells and replicate themselves in tumor cells; upon lysis of infected tumor cells, new virion particles burst out and proceed to infect additional tumor cells. This idea was initially tested in the middle of the last century and merged with renewed ones over the last 30 years due to the technological advances in virology and in the use of viruses as vectors for gene transfer (for the history of oncolytic viruses, see [2]). Oncolytic viruses have shown efficacy in clinical trials [3]. However, the immune response presents a challenge in maximizing efficacy. The major problem is the complexity of the innate and adaptive immune responses in the process of oncolytic viral therapy [4].

Mathematical models have been applied to the understanding of oncolytic virotherapy since fifteen years ago. Wu et al. [5] and Wein et al. [6] proposed and analyzed a system of partial differential equations that is essentially a radially symmetric epidemic model embedded in a Stefan problem to describe some aspect of cancer virotherapy. They were interested in three alternative virus-injection strategies: a fixed fraction of cells preinfected with the virus is introduced throughout the entire tumor volume, within the tumor core, or within the tumor rim. Wodarz [7] and his review paper [8] formulated a simple model with three ordinary differential equations. He studied three hypothetical situations: viral cytotoxicity alone kills tumor cells, a virus-specific lytic CTL response contributes to killing of infected tumor cells, and the virus elicits immunostimulatory signals within the tumor, which promote the development of tumor-specific CTL. Komarova and Wodarz [9] and Wodarz and Komarova [10] analyzed several possible mathematical formulations of oncolytic virus infection in terms of two ordinary differential equations, while Novozhilov et al. [11] analyzed ratio based oncolytic virus infection models. Bajzer et al. [12] used three ordinary differential equations to model specific cancer virotherapy with measles virus, and then they considered optimization of viral doses, number of doses, and timing with a simple mathematical model of three ordinary differential equations for cancer virotherapy [13].

Friedman et al. [14] proposed a free boundary problem with nonlinear partial differential equations to study brain tumor glioma with mutant herpes simplex virus therapy. The model incorporated immunosuppressive agent cyclophosphamide to reduce the effect of the innate immune response. This model reveals the oscillation of cell populations in the process of oncolytic viral therapy. Vasiliu and Tian [15] proposed a simplified model to study the cell population oscillation in oncolytic virotherapy, which may be caused by interaction between infected tumor cells and innate immune cells. To obtain a basic dynamic picture of oncolytic viral therapy, Tian [16] proposed a simple model with three ordinary differential equations to represent interaction among tumor cells, infected tumor cells, and oncolytic viruses and concluded that the viral therapeutic dynamics is largely determined by the viral burst size. To further understand how the viral burst size is affected, Wang et al. [17] and Tian et al. [18] incorporated virus lytic cycle as delay parameter into the basic model. These delay differential equation models gave another explanation of cell population oscillation and revealed a functional relation between the virus burst size and lytic cycle. In a recent paper [19], Choudhury and Nasipuri considered a simple model of three ordinary differential equations for the dynamics of oncolytic virotherapy in the presence of immune response. However, this model did not include the free virus population, and it may not give a complete picture of dynamics of viral therapy with innate immune response.

All proposed mathematical models have given some insights into oncolytic virotherapy. However, there is a considerable need to understand the dynamics of oncolytic virotherapy in the presence of immune responses [4], particularly, to understand the different effects of the innate immune system and adaptive immune system on virotherapy. In response to this call in [4], we plan to construct a comprehensive mathematical model for oncolytic virotherapy with both innate and adaptive immune responses. Toward this end, we will first build a mathematical model for oncolytic virotherapy with the innate immune system based on our basic model proposed in [16]. There are several types of cells that are involved in the innate immune response in virotherapy. So far, the experiments show that natural killer cells, macrophages, and neutrophils have significant effects in viral therapy [4]. For the sake of simplicity, we lump all these innate immune cell types to one variable, the innate immune cell population, in our mathematical model.

Our basic dynamical model for oncolytic virotherapy studied in [16] is as follows:

(1) dxdt=λx1−x+yK−βxv,dydt=βxv−δy,dvdt=bδy−βxv−γv,

where *x* stands for uninfected tumor cells, *y* for infected tumor cells, and *v* for free viruses. For the details of explanations and results, the reader is referred to [16]. The innate immune response reduces infected tumor cells and viruses [4, 14]. We incorporate these effects into the basic model. Denoting the innate immune cell population by *z*, we have the following system:

(2) dxdt=λx1−x+yC−βxv,dydt=βxv−μyz−δy,dvdt=bδy−βxv−kvz−γv,dzdt=syz−ρz,

where *λ* is tumor growth rate, *C* is the carrying capacity of tumor cell population, *β* is the infected rate of the virus, *μ* is immune killing rate of infected tumor cells, *δ* is death rate of infected tumor cells, *b* is the burst size of oncolytic viruses (i.e., the number of new viruses coming out from a lysis of an infected cell), *k* is immune killing rate of viruses, *γ* is clearance rate of viruses, *s* is the stimulation rate of the innate immune system, and *ρ* is immune clearance rate.

In this study, we analyze this four-dimensional system (2). Our analysis and numerical study show that the dynamics of the model is largely determined by the viral burst size *b* and parameters related to the innate immune response. We can denote the dynamical behaviors of the model by *b*, the ratio of the innate immune killing rate of infected tumor cells over the innate immune killing rate of free viruses by *μ*/*k*, the relative innate immune killing rate of viral therapy by *K*, the ratio of the innate immune clearance rate over the stimulation rate of the innate immune system by *ρ*/*Cs*, and the relative innate immune response decay rate by *N*. These two combined parameters are related to the innate immune response. Comparing with the basic model in [16], there are several critical values of the oncolytic viral burst size *b*, *b*_*s*_1__, *b*_*s*_2__, $\bar{b}$, and *b*_0_, where $\bar{b}$ is a function of the relative innate immune killing rate *K* and relative innate immune response decay rate *N*. When *b* is smaller than $\bar{b}$ and the two relative rates are constrained in some intervals, the system has 5 equilibrium solutions and 2 of them are positive, while these two positive equilibrium points coalesce when $b=\bar{b}$. When *b* is greater than *b*_*s*_1__ or $\bar{b}$ and the two relative rates are in the complement intervals, the system has at most 3 equilibrium solutions with 0 innate immune components. An interesting fact is that the equilibrium points where Hopf bifurcations arise for both models, (2) and the one in [16], are corresponding to each other. Therefore, we may conclude that the innate immune response complicates the oncolytic virotherapy in the way of creating more equilibrium solutions when the oncolytic viral burst size is not too big, say less than $\bar{b}$, while the dynamics is similar to the system without the presence of the innate immune response when the oncolytic viral burst size is big.

The rest of this article is organized as follows.  Section 2 presents analysis of model (2) in 4 subsections.  Section 2.1 gives preliminary results about the model, Section 2.2 calculates equilibrium solutions, Section 2.3 studies stability of equilibrium solutions, and Section 2.4 provides bifurcation analysis of the model and the main Theorem 14 to summarize the dynamical behaviors of the model (2).  Section 3 provides a numerical study and discussion, where we numerically compute some dynamical characteristics and simulate the model, and we also compare the dynamics of our model with the basic model of oncolytic virotherapy in [16].

## 2. Analysis of the Mathematical Model

We conduct a detailed analytical study of our proposed model in this section. The major properties of dynamical behaviors of our model are summarized in Theorem 14. For each analysis result, we also provide biological interpretations or implications as appropriate.

### 2.1. Positive Invariant Domain

In order to simplify system (2), we apply nondimensionalization by setting *τ* = *δt*, $x=C\bar{x}$, $y=C\bar{y}$, $v=C\bar{v}$, $z=C\bar{z}$ and rename parameters *r* = *λ*/*δ*, *a* = *Cβ*/*δ*, *c* = *μC*/*δ*, *d* = *kC*/*δ*, *e* = *γ*/*δ*, *m* = *sC*/*δ*, and *n* = *ρ*/*δ*. Then system (2) becomes

(3) dx¯dτ=rx¯1−x¯−y¯−ax¯ v¯,dy¯dτ=ax¯ v¯−cy¯ z¯−y¯,dv¯dτ=by¯−ax¯ v¯−dv¯ z¯−ev¯,dz¯dτ=my¯ z¯−nz¯.

For convenience, dropping all the bars and writing *τ* as *t*, we obtain

(4) dxdt=rx1−x−y−axv,dydt=axv−cyz−y,dvdt=by−axv−dvz−ev,dzdt=myz−nz.

We assume that all parameters are nonnegative.

**Lemma 1 .**

If *x*(0) ≥ 0, *y*(0) ≥ 0, *v*(0) ≥ 0, and *z*(0) ≥ 0, then the solution of system (4) *x*(*t*) ≥ 0, *y*(*t*) ≥ 0, *v*(*t*) ≥ 0, and *z*(*t*) ≥ 0 for *t* ≥ 0. Furthermore, if 0 ≤ *x*(0) + *y*(0) ≤ 1, then 0 ≤ *x*(*t*) + *y*(*t*) ≤ 1 for *t* ≥ 0, and lim sup_*t*→+*∞*_*v*(*t*) ≤ *b*/*e*.

**Proof**

The proof is similar to that of Lemma 3.2 in [16]. Here we only show the second part of this lemma by using comparison theorem for ODEs; that is, if 0 ≤ *x*(0) + *y*(0) ≤ 1, then 0 ≤ *x*(*t*) + *y*(*t*) ≤ 1 and lim sup_*t*→+*∞*_*v*(*t*) ≤ *b*/*e*. In fact, since *x*(*t*), *y*(*t*), and *v*(*t*) are nonnegative for all *t* ≥ 0,

(5) x′t=rx1−x−y−axv≤rx1−x−y≤rx1−x.

As *x*(0) ≤ *x*(0) + *y*(0) ≤ 1, by the comparison theorem we get *x*(*t*) ≤ 1. On the other hand, since

(6) x′t+y′t=rx1−x−y−cyz−y≤rx1−x−y≤r1−x−y,

again by the comparison theorem we also have 0 ≤ *x*(*t*) + *y*(*t*) ≤ 1. It follows that 0 ≤ *y*(*t*) ≤ 1. Then *v*′(*t*) = *by* − *axv* − *dvz* − *ev* ≤ *b* − *ev*, so by the comparison theorem *v*(*t*) ≤ *b*/*e* + *v*(0)exp⁡(−*et*). Taking lim sup both sides yield lim sup_*t*→+*∞*_*v*(*t*) ≤ *b*/*e*.

We then conclude that the positive invariant domain of system (4) is

(7) D=x,y,v,z:x≥0,  y≥0,  v≥0,  z≥0,  0≤x+y≤1.

This is also a biological meaningful range for the variables. We will regard the whole domain *D* as a global domain.

### 2.2. Equilibrium Solutions

We know that the dynamics of oncolytic viral therapy without the presence of the immune response can be characterized by the burst size *b* [16]. The effects of the innate immune system on the virotherapy in our model are encoded in the parameters *c*, *d*, and *m*. To understand how the innate immune system affects the dynamics of oncolytic viral therapy, we use three combined parameters, the viral burst size *b*, the relative immune killing rate *K* = *c*/*d*, and the relative immune response decay rate *N* = *n*/*m*, to describe the solution behaviors of our model. We summarize the possible equilibrium solutions in the following lemma.

**Lemma 2 .**

When (*r*/*a*)(1/*N* − 1) < *K* < 1/(*a* + *e* − *aN*), $N<1+(1/2)\left(e/a+1/r-\sqrt{(e/a+1/r{)}^{2}+4/r}\right)$, and $b>\bar{b}$ with *b* > 1 + *e*/*a*, system (4) has 3 equilibrium solutions: *E*_0_, *E*_1_, and *E*_2_. When either *K* ≤ (*r*/*a*)(1/*N* − 1) or *K* > 1/(*a* + *e* − *aN*) and $b=\bar{b}$ with *g*(*K*) > 0, system (4) has a unique positive equilibrium solution: *E*_3_. When either *K* ≤ (*r*/*a*)(1/*N* − 1) or *K* > 1/(*a* + *e* − *aN*) and $b<\bar{b}$ with *g*(*K*) > 0, system (4) has two distinct positive equilibrium solutions: *E*_4_ and *E*_5_.

In what follows we will analyze the existence of equilibrium solutions. First, let *X* = (*x*, *y*, *v*, *z*)^*T*^ and

(8) FX=rx1−x−y−axv,axv−cyz−y,by−axv−dvz−ev,myz−nzT.

Then system (4) can be simply written as the autonomous system *dX*/*dt* = *F*(*X*). We assume that (*x*, *y*, *v*, *z*) ∈ *D*. The equilibrium points are solutions of the equation *F*(*X*) = 0; that is,

(9) xr1−x−y−av=0,axv=ycz+1,by=vax+dz+e,zmy−n=0.

If *x* = 0, then, from the second and the third equation of (9), *y*(*cz* + 1) = 0 and *by* = *v*(*dz* + *e*). Since *cz* + 1 > 0, then *y* = 0. It leads to *v*(*dz* + *e*) = 0, which implies *v* = 0. The last equation of (9) gives −*nz* = 0, which implies *z* = 0. Thus *E*_0_ = (0,0, 0,0) is an equilibrium point.

If *x* ≠ 0, the first equation of (9) implies *r*(1 − *x* − *y*) = *av*. Consider the last one *z*(*my* − *n*) = 0. If *z* = 0, from the second and the third equation of (9), we get *axv* = *y* and *by* = *v*(*ax* + *e*). These follow *abxv* = *v*(*ax* + *e*) and hence *v*(*abx* − *ax* − *e*) = 0. If *v* = 0, then *y* = 0 and *r*(1 − *x*) = 0, which implies that *x* = 1. So *E*_1_ = (1,0, 0,0) is an equilibrium point.

Now if *v* ≠ 0, then *a*(*b* − 1)*x* = *e*. Since we want to find positive equilibrium points, we assume *b* > 1. Then *x* = *e*/*a*(*b* − 1). From the equation *r*(1 − *x* − *y*) = *av*, we have *rx*(1 − *x*) = *rxy* + *axv* = *rxy* + *y* = *y*(1 + *rx*). It follows that

(10) $$\begin{array}{c} y=\frac{rx\left(1-x\right)}{1+rx}=\frac{re\left(ab-a-e\right)}{a\left(b-1\right)\left(ab-a+re\right)}. \end{array}$$

Since *axv* = *y*, we have *v* = *y*/*ax* = *r*(*ab* − *a* − *e*)/*a*(*ab* − *a* + *re*). Thus we get the third critical point

(11) E2=eab−1,reab−a−eab−1ab−a+re,rab−a−eaab−a+re,0.

Notice that in order for the first three coordinates of *E*_2_ to be positive, it is enough that *ab* − *a* − *e* > 0; that is, *b* > *e*/*a* + 1.

Next, if *z* ≠ 0, then *y* = *n*/*m*. Set *N* = *n*/*m*, then *y* = *N*. From the equation *r*(1 − *x* − *y*) = *av*, we can derive *x* = 1 − *N* − *av*/*r*. By the third equation of (9), *z* = ((*b* − 1)*N* − *ev*)/(*cN* + *dv*). Plugging these expressions into the second equation gives *f*(*v*)≕*v*^3^ + *a*_2_*v*^2^ + *a*_1_*v* + *a*_0_ = 0, where

(12) a2=cdN+raN−1,a1=rNa2·cd−a+aN−e+dc,a0=b·rN2a2·cd.

Since *f*(0) = *a*_0_ > 0 and lim_*v*→−*∞*_*f*(*v*) = −*∞*, *f*(*v*) has at least one negative root, say *v*_1_ < 0. Assume that *v*_1_ is the least root. If *a*_1_ > 0 and *a*_2_ > 0, then *f* has no sign changes at all and hence the other two roots of *f* are either both negative or both complex. Notice that *a*_1_ > 0 and *a*_2_ > 0 are equivalent to (*r*/*a*)(1/*N* − 1) < *c*/*d* < 1/(*a* + *e* − *aN*) and $N<1+(1/2)\left(e/a+1/r-\sqrt{(e/a+1/r{)}^{2}+4/r}\right)$. In this case, the system only has 3 equilibrium points: *E*_0_, *E*_1_, and *E*_2_.

We now look at other situations of *f*(*v*). Taking derivative, *f*′(*v*) = 3*v*^2^ + 2*a*_2_*v* + *a*_1_. By long division,

(13) fv=13v+a29f′v+293a1−a22v−19a1a2+a0.

Suppose that *a*_2_^2^ − 3*a*_1_ > 0, then *f*′ has 2 distinct roots, *v*_1_^*∗*^ and *v*_2_^*∗*^, where $v_{2}^{*}=\left(-a_{2}+\sqrt{a_{2}^{2}-3a_{1}}\right)/3≕A$. If *v*_2_^*∗*^ > 0 and *f*(*v*_2_^*∗*^) = 0, then *f* has one negative root, *v*_1_, and one positive root, *v*_2_ = *v*_3_ = *v*_2_^*∗*^ = (9*a*_0_ − *a*_1_*a*_2_)/2(*a*_2_^2^ − 3*a*_1_). In this case, in addition to the 3 equilibrium points *E*_0_, *E*_1_, and *E*_2_, system (4) has one positive equilibrium point: *E*_3_ = (1 − *N* − *aA*/*r*, *N*, *A*, ((*b* − 1)*N* − *eA*)/(*cN* + *dA*)). To guarantee all four coordinates of *E*_3_ are positive, we need to impose 1 − *N* − *aA*/*r* > 0 and (*b* − 1)*N* − *eA* > 0, which imply that *v*_2_^*∗*^ = *A* < *u*^*∗*^ = min⁡{(1 − *N*)(*r*/*a*), ((*b* − 1)/*e*)*N*}.

On the other hand, if *v*_2_^*∗*^ > 0 and *f*(*v*_2_^*∗*^) < 0, then *f* has one negative root, *v*_1_, and 2 distinct positive roots, 0 < *v*_2_ < *v*_2_^*∗*^ < *v*_3_ < *u*^*∗*^. Hence system (4) has two positive equilibrium points: *E*_4,5_ = (1 − *N* − *av*_2,3_/*r*, *N*, *v*_2,3_, ((*b* − 1)*N* − *ev*_2,3_)/(*cN* + *dv*_2,3_)). Notice that *v*_2_^*∗*^ = *A* > 0 is equivalent to either *a*_2_ ≤ 0, *a*_2_^2^ − 3*a*_1_ > 0 or *a*_2_ > 0 > *a*_1_. Furthermore, *a*_2_ ≤ 0, *a*_2_^2^ − 3*a*_1_ > 0 are equivalent to *c*/*d* ≤ (*r*/*a*)(1/*N* − 1) and *g*(*c*/*d*) > 0, where *g*(*x*) = *x*^2^ + (*r*/*aN* − *r*/*a* + 3*re*/*a*^2^*N*)*x* + (*r*^2^/*a*^2^)(1/*N* − 1)^2^ − 3*r*/*a*^2^*N*; *a*_2_ > 0 > *a*_1_ is equivalent to *c*/*d* > max⁡{(*r*/*a*)(1/*N* − 1), 1/(*a* + *e* − *aN*)}. The condition *f*(*v*_2_^*∗*^) ≤ 0 is equivalent to *v*_2_^*∗*^ ≥ (9*a*_0_ − *a*_1_*a*_2_)/2(*a*_2_^2^ − 3*a*_1_). That is, $\left(-a_{2}+\sqrt{a_{2}^{2}-3a_{1}}\right)/3\geq \left(9a_{0}-a_{1}a_{2}\right)/2(a_{2}^{2}-3a_{1})$, and we have $\bar{b}≔a^{2}d/rcN^{2}\cdot \left(-2a_{2}^{3}+9a_{1}a_{2}+2(a_{2}^{2}-3a_{1}{)}^{3/2}\right)/27\geq b$. The critical value $\bar{b}$ is important for describing the dynamics of system (4).

We summarize the details of the analysis above as follows.

(i) When *x* = 0, we have equilibrium solution *E*_0_ = (0,0, 0,0).
(ii) When *x* ≠ 0, we have the following cases.
  (a) If *z* = 0, then
    (1) when *v* = 0, we have equilibrium solution *E*_1_ = (1,0, 0,0).
    (2) when *v* ≠ 0 and *b* > 1 + *e*/*a*, we have
      (14) E2=eab−1,reab−a−eab−1ab−a+re,rab−a−eaab−a+re,0.
  (b) If *z* ≠ 0, then *x* = 1 − *N* − (*a*/*r*)*v*, *y* = *N*, *z* = ((*b* − 1)*N* − *ev*)/(*cN* + *dv*), and *v* satisfies the following cubic equation:
    (15) $$\begin{array}{c} v^{3}+a_{2}v^{2}+a_{1}v+a_{0}=0, \end{array}$$
  where *a*_2_ = (*c*/*d*)*N* + (*r*/*a*)(*N* − 1), *a*_1_ = *rN*/*a*^2^ · (*c*/*d*)(−*a* + *aN* − *e* + *d*/*c*), *a*_0_ = *b* · *rN*^2^/*a*^2^ · *c*/*d*.
  In this case, we can conclude the following.
    (1) If *K* ∈ ((*r*/*a*)(1/*N* − 1), 1/(*a* + *e* − *aN*)), $N<1+(1/2)\left(e/a+1/r-\sqrt{(e/a+1/r{)}^{2}+4/r}\right)$, and *b* > 1 + *e*/*a*, then system (4) has three equilibrium points: *E*_0_, *E*_1_, and *E*_2_.
    (2) If either *K* ≤ (*r*/*a*)(1/*N* − 1), *g*(*K*) > 0, or *K* > 1/(*a* + *e* − *aN*) and $b=\bar{b}$, then system (4) has a unique positive equilibrium point:
      (16) $$\begin{array}{c} E_{3}=\left(\;1-N-\frac{aA}{r},N,A,\frac{\left(b-1\right)N-eA}{cN+dA}\;\right), \end{array}$$
    where *v*_2_^*∗*^ = *A* < *u*^*∗*^≔min⁡{(1 − *N*)(*r*/*a*), (*b* − 1)*N*/*e*} and
      (17) gx=x2+raN−ra+3rea2Nx+r2a21N−12−3ra2N.
    (3) If either *K* ≤ (*r*/*a*)(1/*N* − 1), *g*(*K*) > 0, or *K* > 1/(*a* + *e* − *aN*) and $b<\bar{b}$, then system (4) has two distinct positive equilibrium points:
      (18) E4=1−N−v2q,N,v2,b−1N−ev2cN+dv2,E5=1−N−v3q,N,v3,b−1N−ev3cN+dv3,
    where *v*_2_ and *v*_3_ are two distinct positive real roots of the above cubic equation that satisfy 0 < *v*_2_ < *v*_2_^*∗*^ < *v*_3_ < *u*^*∗*^, in which $v_{2}^{*}=A≔\left(-a_{2}+\sqrt{a_{2}^{2}-3a_{1}}\right)/3$.

In order to interpret our mathematical conditions biologically, we need to understand the combined parameters *N* and *K* first. *N* = *ρ*/*sC* can be considered as a relative immune response decay rate since *ρ* is innate immune cell death rate, *s* is innate immune stimulating rate by infection, and *C* is tumor carrying capacity. *K* = *c*/*d* = *μ*/*k* is the ratio of the rate of immune killing infected tumor cells over the rate of immune killing viruses, which can be considered as a relative immune killing rate of viral therapy since it describes the ability of the innate immune system destroying infection versus destroying viruses. Biological interpretation of Lemma 2 is as follows. If there are no tumor cells, we have zero equilibrium *E*_0_. If we do not consider the effect of the immune system, and the viruses are not powerful, that is, the burst size is smaller than a critical value, then the system has the equilibrium *E*_1_ with only tumor cells; if the viruses are powerful, that is, the burst size is greater than a critical value, then the system has the equilibrium *E*_2_ with the coexistence of tumor cells, infected tumor cells, and viruses. When we consider the immune effect, if the burst size is another critical value $\bar{b}$ and the relative immune killing rate satisfies some conditions, the system has a unique positive equilibrium; if the burst size is greater than that critical value and the relative immune killing rate satisfies certain similar conditions, the system has other two positive equilibria. Combining stability analysis, we can have more biological implications in the next two subsections.

### 2.3. Stability Analysis of Equilibrium Solutions

In this subsection, we apply various methods to analyze the asymptotically stable behaviors of equilibrium solutions. By finding the eigenvalues of the variational matrix of system (4) at the equilibrium points, we show *E*_0_ is locally unstable, *E*_1_ is locally asymptotically stable if *b* < 1 + *e*/*a* and unstable if *b* > 1 + *e*/*a*, and *E*_2_ is locally asymptotically stable if *b* is in some range, while *E*_3_, *E*_4_, and *E*_5_ are locally unstable when *b*, *K*, and *N* satisfy some conditions. We use Lyapunov functions to show *E*_1_ is globally asymptotically stable if *b* < 1 + *e*/*a* and *N* > 1. We apply the center manifold theorem to show *E*_1_ is locally asymptotically stable if *b* = 1 + *e*/*a*. We summarize the main results in Lemma 3. For the combined parameter values, *b*_*s*_*i*__, *i* = 1,2, *J*, ${\bar{b}}_{j},\;\;j=1,2,3$, they will be defined in the following context. For methods we applied in this subsection, we refer to Allen [20] for basic knowledge of stability analysis and Carr [21] for center manifolds.

**Lemma 3 .**

*E*_0_ is unstable. *E*_1_ is globally asymptotically stable when *b* < 1 + *e*/*a* and *N* > 1 and unstable when *b* > 1 + *e*/*a*. *E*_2_ is locally asymptotically stable when *b* ∈ (*b*_*s*_1__, *b*_*s*_2__)∩*J*. *E*_3_ is unstable when $\bar{b}<{\bar{b}}_{1}$. *E*_4_ and *E*_5_ are unstable when $\bar{b}<{\bar{b}}_{2,3}$.

We look at the stability of trivial equilibrium solutions first. The variational matrix of system (4) is given by

(19) ∂F∂X=r−2xr−ry−av−rx−ax0av−cz−1ax−cy−avb−ax−dz−e−dv0mz0my−n.

At the critical point *E*_0_, the variational matrix is

(20) $$\begin{array}{c} \left(\begin{array}{cccc} r & 0 & 0 & 0\\ 0 & -1 & 0 & 0\\ 0 & b & -e & 0\\ 0 & 0 & 0 & -n \end{array}\right). \end{array}$$

The corresponding eigenvalues are *r*, −1, −*e*, and −*n*. We know that the local stability of *E*_0_ of system (4) is the same as that of the linearized system at *E*_0_. Since *r* > 0, *E*_0_ is locally unstable. For system (4), the local stable invariant manifold is tangent to the *y*-*v*-*z* subspace, and the unstable invariant manifold is tangent to the *x*-axis. Biologically, the tumor will grow from an initial small value around *E*_0_ without viruses and infected tumor cells.

**Proposition 4 .**

The equilibrium solution *E*_1_ is locally asymptotically stable when *b* < 1 + *e*/*a*, and it is locally unstable when *b* > 1 + *e*/*a*. *E*_1_ is globally asymptotically stable when *b* < 1 + *e*/*a* and *N* > 1.

**Proof**

At the equilibrium point *E*_1_, the variational matrix is

(21) $$\begin{array}{c} \frac{\partial F}{\partial X}\left(\;E_{1}\;\right)=\left(\begin{array}{cccc} -r & -r & -a & 0\\ 0 & -1 & a & 0\\ 0 & b & -a-e & 0\\ 0 & 0 & 0 & -n \end{array}\right). \end{array}$$

The characteristic polynomial of this matrix is (*λ* + *n*)(*λ* + *r*)(*λ*^2^ + (1 + *a* + *e*)*λ* + *a* + *e* − *ab*). The eigenvalues are *λ*_1_ = −*r*, *λ*_2_ = −*n*, and $\lambda_{3,4}=(1/2)\left(-1-a-e\pm \sqrt{(1-a-e{)}^{2}+4ab}\right)$. Since the eigenvalues *λ*_1_ = −*r*, *λ*_2_ = −*n*, and $\lambda_{3}=(1/2)\left(-1-a-e-\sqrt{(1-a-e{)}^{2}+4ab}\right)$ are negative for all positive parameters, *E*_1_ is locally asymptotically stable if and only if *λ*_4_ < 0. This is equivalent to $\sqrt{(1-a-e{)}^{2}+4ab}<1+a+e$, which is the same as *b* < 1 + *e*/*a*. Similarly, if *b* > 1 + *e*/*a*, then $\lambda_{4}=(1/2)\left(-1-a-e+\sqrt{(1-a-e{)}^{2}+4ab}\right)>0$, and hence *E*_1_ is unstable.

In fact, we can show that *E*_1_ is globally asymptotically stable in the positive invariant domain *D* when *b* < 1 + *e*/*a* and *m* < *n* by constructing two Lyapunov functions according to different ranges of the parameter *b*. For convenience, we translate *E*_1_ into the origin by setting $x=1-\bar{x}$, $y=\bar{y}$, $v=\bar{v}$, and $z=\bar{z}$. After dropping all the bars over variables, system (4) becomes

(22) dxdt=−rx+ry+av+rx2−rxy−axv,dydt=av−axv−cyz−y,dvdt=by+axv−dvz−a+ev,dzdt=myz−nz,

while the domain *D* is translated to *D*_1_ = {(*x*, *y*, *v*, *z*) : 0 ≤ *x* ≤ 1, *y* ≥ 0, *v* ≥ 0, *z* ≥ 0, 0 ≤ *x* − *y* ≤ 1}. For any initial condition (*x*_0_, *y*_0_, *v*_0_, *z*_0_) ∈ *D*_1_, the solution of (22) satisfies 0 ≤ *x*(*t*) ≤ 1, 0 ≤ *y*(*t*) ≤ 1, *v*(*t*) ≥ 0, and *z*(*t*) ≥ 0. Now we construct two Lyapunov functions corresponding to the values of the parameter *b* to prove *y*(*t*) and *v*(*t*) approach 0, then we show *x*(*t*) and *z*(*t*) also tend to 0.

When 0 < *b* < 1, we define the Lyapunov function *V*_1_(*x*, *y*, *v*, *z*) = *y* + *v*. It is clear that *V*_1_(*x*, *y*, *v*, *z*) > 0, and the orbital derivative *dV*_1_/*dt* = *dy*/*dt* + *dv*/*dt* = (*b* − 1)*y* − *cyz* − *dvz* − *ev* < 0. When 1 ≤ *b* < 1 + *e*/*a*, consider the Lyapunov function *V*_2_(*x*, *y*, *v*, *z*) = (1/2)*ab*(*a* + *e*)*y*^2^ + *a*^2^*byv* + (1/2)*a*^2^*v*^2^. Obviously, *V*_2_(*x*, *y*, *v*, *z*) > 0. The orbital derivative along a solution is given by

(23) dV2dt=aba+eydydt+a2bdydtv+a2bydvdt+a2vdvdt=aba+eyav−axv−cyz−y+a2bav−axv−cyz−yv+a2byby+axv−dvz−av−ev+a2vby+axv−dvz−av−ev=aby2+a2v2ab−a−e+a3xv21−b−a2bcxyv−abca+ey2z−a2byvzc+d−a2dv2z.

1 ≤ *b* < 1 + *e*/*a*; that is, *ab* − *a* − *e* < 0 and 1 − *b* ≤ 0; therefore *dV*_2_/*dt* < 0. Combining these two Lyapunov functions gives *y*(*t*) → 0 and *v*(*t*) → 0 as *t* → *∞* when *b* < 1 + *e*/*a*. Considering the component *x*(*t*), we have

(24) dxdt=−rx+ry+av+rx2−rxy−axv=1−xry+av−rx≤ry+av−rx.

By the comparison theorem,

(25) $$\begin{array}{c} 0\leq x\left(\;t\;\right)\leq x\left(\;0\;\right)e^{-rt}+e^{-rt}\overset{t}{\underset{0}{\int }}\left(\;ry\left(\;s\;\right)+av\left(\;s\;\right)\;\right)e^{rs}ds. \end{array}$$

Taking limit of both sides as *t* → *∞* and using the L'Hospital's Rule and the fact that *y*(*t*) and *v*(*t*) approach 0 yield *x*(*t*) → 0. Finally, since *y*(*t*) ≤ 1, we have *dz*/*dt* ≤ (*m* − *n*)*z*. By the comparison theorem, 0 ≤ *z*(*t*) ≤ *z*(0)*e*^(*m*−*n*)*t*^ → 0 as *t* → *∞*, since *m* − *n* < 0. Therefore system (3) has a global attractor *E*_1_.

Biologically, Proposition 4 is easy to understand. When the viral burst size is smaller than a critical value which is *b* = 1 + *e*/*a*, there will not be enough newly produced viruses to infect tumor cells. The therapy fails. The model system will be stable in the state of tumor cells and free of infected tumor cells, viruses, and immune cells.  Proposition 5 ensures mathematically that this critical burst size is the smallest one that will make the virotherapy completely fail. One obvious medical implication is that we have to genetically increase the viral burst size in order to have effective virotherapy.

We next consider the stability of *E*_1_ when *b* = 1 + *e*/*a*. This is a critical case, since the linearized system at *E*_1_ has three negative eigenvalues and one zero eigenvalue. we have to reduce the system to its local center manifold. We actually have the following proposition.

**Proposition 5 .**

The equilibrium solution *E*_1_ is locally asymptotically stable when *b* = 1 + *e*/*a*.

**Proof**

Consider *b* = 1 + *e*/*a*, which implies that *ab* = *a* + *e*. The linearized system at *E*_1_ has three negative eigenvalues and one zero eigenvalue. In order to determine the stability of *E*_1_, we use the center manifold theorem to reduce system (22) into a center manifold, and then we study the reduced system. So we separate it into two parts, one with zero eigenvalue and the other with negative eigenvalues. The matrix corresponding to the linear part of system (22) is

(26) $$\begin{array}{c} L=\left(\begin{array}{cccc} -r & r & a & 0\\ 0 & -1 & a & 0\\ 0 & b & -ab & 0\\ 0 & 0 & 0 & -n \end{array}\right), \end{array}$$

which has eigenvalues −*r*, −(1 + *ab*), 0, and −*n*. The associated eigenvectors of these eigenvalues, respectively, are *V*_1_^*T*^ = (1,0, 0,0), *V*_2_^*T*^ = (*ab* − *r*, 1 + *ab* − *r*, −*b*(1 + *ab* − *r*), 0), *V*_3_^*T*^ = (*ar* + *a*, *ar*, *r*, 0), and *V*_4_^*T*^ = (0,0, 0,1). System (22) can be written as *dX*/*dt* = *LX* + *F*, where *F* = (*rx*^2^ − *rxy* − *axv*, −*axv* − *cyz*, *axv* − *dvz*, *myz*)^*T*^. Set *T* = (*V*_1_, *V*_2_, *V*_3_, *V*_4_) and *X* = *TY*; then

(27) $$\begin{array}{c} \frac{dY}{dt}=T^{-1}LTY+T^{-1}F, \end{array}$$

where

(28) $$\begin{array}{c} T^{-1}LT=\left(\begin{array}{cccc} -r & 0 & 0 & 0\\ 0 & -1-ab & 0 & 0\\ 0 & 0 & 0 & 0\\ 0 & 0 & 0 & -n \end{array}\right), \end{array}$$

and *Y* = (*y*_1_, *y*_2_, *y*_3_, *y*_4_)^*T*^ is determined by

(29) x=y1+ab−ry2+ar+ay3,y=1+ab−ry2+ary3,v=−b1+ab−ry2+ry3,z=y4.

Denote *T*^−1^*F* = (*f*_1_, *f*_2_, *f*_3_, *f*_4_)^*T*^; then we can express *f*_*i*_, *i* = 1,2, 3,4, in terms of *y*_*i*_:

(30) f1=rx2−rxy−axv+ab+rab−r21+ab−rraxv+cyz+a1+ab−rrdvz−axv=A11y12+A12y1y2+A13y1y3+A22y22+A23y2y3+A33y32+A24y2y4+A34y3y4,f2=−1ab+11+ab−raxv+cyz+aab+11+ab−rdvz−axv=B12y1y2+B13y1y3+B22y22+B23y2y3+B33y32,f3=−b1+abraxv+cyz+1ab+1raxv−dvz=C12y1y2+C13y1y3+C22y22+C23y2y3+C33y32,f4=myz=D24y2y4+D34y3y4,

where *A*_*ij*_, *B*_*ij*_, *C*_*ij*_, and *D*_*ij*_ are coefficients that can be easily determined. The transformed system can be expressed as

(31) dZdt=BZ+f1f2f4,dy3dt=Ay3+f3,

where

(32) B=−r000−1−ab000−n,A=0,Z=y1,y2,y4T.

It is easy to check that each *f*_*i*_, *i* = 1,2, 3,4, is twice differentiable function, *f*_*i*_(0,0, 0,0) = 0 and *Df*_*i*_(0,0, 0,0) = 0, where *Df*_*i*_ is the Jacobian matrix of the function *f*_*i*_. By the center manifold theorem, there exists a center manifold:

(33) Z=hy3,or  y1y2y4=h1y3h2y3h4y3

with *h*(0) = 0 and *Dh*(0) = 0, and it satisfies

(34) Bhy3+f1hy3,y3f2hy3,y3f4hy3,y3=Dhy3·f3hy3,y3.

Since *h*(0) = 0 and *Dh*(0) = 0, we can assume that

(35) hu=h1uh2uh4u=a2u2+a3u3+a4u4+Ou5b2u2+b3u3+b4u4+Ou5d2u2+d3u3+d4u4+Ou5;

here we use the variable *u* instead of *y*_3_ for simplicity. Then we can compute

(36) f1hu,u=f1h1u,h2u,u,h4u=A33u2+Ou3,f2hu,u=f2h1u,h2u,u,h4u=B33u2+Ou3,f3hu,u=f3h1u,h2u,u,h4u=C33u2+Ou3,f4hu,u=f4h1u,h2u,u,h4u=D24b2d2u4+b2d3+b3d2u5+D34d2u3+d3u4+d4u5+Ou6.

By substituting *f*_*i*_'s into (34) and equating both sides of the equation, we can get *C*_33_ = −*a*^2^(*r* + 1)(*b* − 1)/(*ab* + 1) < 0, since *b* = 1 + *e*/*a* > 1. The asymptotically behavior of zero solution of system (31) is governed by that of the equation *dy*_3_/*dt* = *f*_3_(*h*(*y*_3_), *y*_3_) or *dy*_3_/*dt* = *C*_33_*y*_3_^2^ + *O*(*y*_3_^3^). Since *C*_33_ < 0, *y*_3_ = 0 is locally asymptotically stable. Therefore, the trivial solution of system (22) is locally asymptotically stable.

We next consider the stability of the equilibrium solution $E_{2}=(\bar{x},\bar{y},\bar{v},\bar{z})$, where $\bar{x}=e/a(b-1)$, $\bar{y}=re(ab-a-e)/a(b-1)(ab-a+re)$, $\bar{v}=r(ab-a-e)/a(ab-a+re)$, and $\bar{z}=0$. For lately defined *b*_*s*_*i*__ and *J*, we have a proposition as follows.

**Proposition 6 .**

When *b* ∈ (*b*_*s*_1__, *b*_*s*_2__)∩*J* ≠ *∅*, *E*_2_ is locally asymptotically stable.

We show this proposition as follows. The variational matrix at *E*_2_ is given by

(37) L=∂F∂XE2=−reab−1−reab−1−eb−10rab−a−eab−a+re−1eb−1−cy¯−rab−a−eab−a+reb−beb−1−dv¯000my¯−n.

The characteristic polynomial of *L* is

(38) $$\begin{array}{c} p\left(\;\lambda \;\right)=\left|\;\lambda I-L\;\right|=\left[\;\lambda -\left(\;m\bar{y}-n\;\right)\;\right]q\left(\;\lambda \;\right), \end{array}$$

where *q*(*λ*) = *λ*^3^ + *a*_1_*λ*^2^ + *a*_2_*λ* + *a*_3_, and

(39) a1=re+ab−a+abeab−1,a2=rebe+b−1ab−12+reab−a−er−aab−1ab−a+re,a3=reab−a−eab−1.

By Routh-Hurwitz Criterion, all roots of *q*(*λ*) have negative real parts if and only if

(40) H1=a1>0,H2=a1a31a2>0,H3=a1a301a200a1a3>0.

Since *b* > *b*_*s*_1__≕1 + *e*/*a*, *a*_1_ > 0 and *a*_3_ > 0. And those conditions in (40) are equivalent to *H*_2_ = *a*_1_*a*_2_ − *a*_3_ > 0. This inequality is the same as

(41) φb=ab−1ab−a+reab−a+re+abe−be+b−1ab−a+reb−1ab−a−e<r−a.

Therefore, we can conclude that if $\bar{y}<N$ and *φ*(*b*) < *r* − *a*; then *E*_2_ is locally asymptotically stable. Now we can refine this result by considering *H*(*b*) = *H*_2_, and

(42) Φx=−a3x4+a23e+e2+r−a−ae+1x3+ae3re+3a+re2+3ae+r+r2−a2x2+e23ar+2aer+r2e+2a2x+re3r+a.

It is easy to check that *H*(*b*) = *re*Φ(*b* − 1)/*a*^2^(*b* − 1)^3^(*ab* − *a* + *re*). Since *b* > 1 + *e*/*a*, *H*(*b*) and Φ(*b* − 1) have the same roots. Since Φ(*e*/*a*) = *e*^3^(1 + *r* + *e* + *a*)(1 + *e* + *a*)((1 + *r*)/*a*) > 0 and lim_*x*→±*∞*_Φ(*x*) = −*∞*, there are at least one *x*_1_ < *e*/*a* and one *x*_2_ > *e*/*a* so that Φ(*x*_1_) = Φ(*x*_2_) = 0. Then *H*(*b*) has at least one root 1 + *x*_1_ < 1 + *e*/*a* = *b*_*s*_1__ and one root 1 + *x*_2_ > 1 + *e*/*a* = *b*_*s*_1__. We consider three different cases as follows.

- If Φ has 4 distinct real roots, then either 3 roots are bigger than *e*/*a* or only 1 root is bigger than *e*/*a*.
- If Φ has 3 distinct real roots, then one root must be repeated.
- If Φ has 2 distinct real roots, then one root must be bigger than *e*/*a*.

In all cases, we always have at least one root bigger than *e*/*a*; denote it by *x*_0_. Then *x*_0_ > *e*/*a*, Φ(*x*_0_) = 0, and Φ′(*x*_0_) < 0. Let *b*_0_ = 1 + *x*_0_; since

(43) $$\begin{array}{c} H^{\prime }\left(\;x\;\right)=\frac{re}{a^{2}}\cdot \frac{\left(x-1\right)\left(ax-a+re\right)\Phi^{\prime }\left(x-1\right)-\left(4ax-4a+3re\right)\Phi \left(x-1\right)}{{\left(x-1\right)}^{4}{\left(ax-a+re\right)}^{2}}, \end{array}$$

we get *H*′(*b*_0_) = *H*′(1 + *x*_0_) = (*re*/*a*^2^)(Φ′(*x*_0_)/*x*_0_^3^(*ax*_0_ + *re*)) < 0. As *H*′(*b*) is continuous, there is *δ*_1_ > 0 that can be made smaller than *b*_0_ − *b*_*s*_1__ so that *H*′(*b*) < 0 in (*b*_0_ − *δ*_1_, *b*_0_ + *δ*_1_). Thus *H*(*b*) is monotonically decreasing in this interval. Thus, we have proved the following property.

**Property 7 .**

The function *H*(*b*) has at least 2 real roots, one of which is bigger than *b*_*s*_1__ = 1 + *e*/*a*, and the other is less than *b*_*s*_1__. Among all roots bigger than *b*_*s*_1__, there is a root *b*_0_ and a neighborhood of *b*_0_, (*b*_0_ − *δ*_1_, *b*_0_ + *δ*_1_) where *δ*_1_ < *b*_0_ − *b*_*s*_1__ so that *H*′(*b*_0_) < 0 and *H*(*b*) is decreasing in this interval.

Define *I*_*p*_ = {*b* > *b*_*s*_1__ : *H*(*b*) > 0}, *I*_*n*_ = {*b* > *b*_*s*_1__ : *H*(*b*) < 0}, and *I*_0_ = {*b* > *b*_*s*_1__ : *H*(*b*) = 0}. All these sets are nonempty and *I*_0_ has either at least 1 element or at most 3 elements. Let *b*_*s*_2__ = min⁡*I*_0_. Note that *b*_0_ ≥ *b*_*s*_2__. It is easy to check that when *b* ∈ (*b*_*s*_1__, *b*_*s*_2__), *H*(*b*) > 0.

Next, we refine the condition $\bar{y}<N$, where *N* = *n*/*m*. This inequality is equivalent to

(44) $$\begin{array}{c} N{\left(\;a\left(\;b-1\;\right)\;\right)}^{2}+re\left(\;N-1\;\right)\left(\;a\left(\;b-1\;\right)\;\right)+re^{2}>0. \end{array}$$

Considering it as a quadratic polynomial of *a*(*b* − 1), we have Δ = *re*^2^[*r*(*N* − 1)^2^ − 4*N*]. If Δ < 0, then this inequality is always true for all *N*, so $\bar{y}<N$ is always true. If Δ ≥ 0, then

- when *N* ≥ 1, the right-hand side of the inequality has 2 negative roots *N*_1_ < *N*_2_ < 0, but since *N* > 0, this inequality is obviously true and hence $\bar{y}<N$ is always true;
- when *N* < 1, the inequality is equivalent to *b* ∈ (1,1 + *N*_1_/*a*)∪(1 + *N*_2_/*a*, +*∞*), where *N*_1_ and *N*_2_ are 2 positive roots of the right-hand side of the inequality (they may be equal).

Let *J* = (1,1 + *N*_1_/*a*)∪(1 + *N*_2_/*a*, +*∞*). Then we have the following result: if (*b*_*s*_1__, *b*_*s*_2__)∩*J* ≠ *∅* and *b* is in this intersection, then *E*_2_ is locally asymptotically stable.

Biologically, when the viral burst size is becoming larger and between two critical values, Proposition 6 says that the virotherapy will reach a stable state which is free of innate immune cells. It is reasonable that these two critical burst sizes are related to the relative immune response decay rate. Actually, in order to have this equilibrium, it requires that the relative immune response decay rate is small. In other words, when the relative immune response decay rate is small and the viral burst size is becoming larger, the virotherapy can have a partial success where the innate immune system has no effects on the therapy, and tumor cells, infected tumor cells, and viruses coexist.

For positive equilibrium solutions *E*_3_, *E*_4_, and *E*_5_, when they exist, we derive conditions under which they are unstable.

**Proposition 8 .**

*E*_3_ is locally unstable when $\bar{b}<{\bar{b}}_{1}$. *E*_4_ and *E*_5_ are locally unstable when $\bar{b}<{\bar{b}}_{2,3}$.

**Proof**

When *f*(*v*) = 0 has a unique positive root *v*_2_ = *v*_3_ = *v*_2_^*∗*^ = *A* < *u*^*∗*^, the system has a unique positive equilibrium solution *E*_3_ = (1 − *N* − *aA*/*r*, *N*, *A*, ((*b* − 1)*N* − *eA*)/(*cN* + *dA*)). The variational matrix at *E*_3_ is

(45) $$\begin{array}{c} \frac{\partial F}{\partial X}\left(\;E_{3}\;\right)=\left(\begin{array}{cccc} -r+rN+aA & -r+rN+aA & -a+aN+\frac{a^{2}A}{r} & 0\\ aA & \frac{-bcN+\left(ce-d\right)A}{cN+dA} & a-aN-\frac{a^{2}A}{r} & -cN\\ -aA & b & -a+aN+\frac{a^{2}A}{r}+\frac{\left(d-db-ce\right)N}{cN+dA} & -dA\\ 0 & m\frac{\left(b-1\right)N-eA}{cN+dA} & 0 & 0 \end{array}\right). \end{array}$$

The characteristic polynomial of this matrix is computed as the quartic polynomial *p*(*λ*) = *λ*^4^ + *b*_3_*λ*^3^ + *b*_2_*λ*^2^ + *b*_1_*λ* + *b*_0_, where

(46) b0=−mb−1N−eAcN+dAr−rN−aA·−a+aN+a2ArcN+dA−cNdb+ce−dNcN+dA+aAcN+dA·a−aN−a2Ar.

Assume that *p*(0) = *b*_0_ < 0. Since lim_*λ*→*∞*_*p*(*λ*) = *∞*, *p*(*λ*) has at least one positive root. The fact *b*_0_ < 0 is equivalent to *b* < ((*cN* + *dA*)^2^/*cdN*^2^)((2*a*^2^/*r*)*A* − *a* + *aN*) − *ce*/*d* + 1. On the other hand, we can compute *b* in terms of coefficients of *f*(*v*) and note that the coefficients *a*_1_, *a*_2_ do not depend on *b*. Since *f*(*v*_2_^*∗*^) = 0, we have *v*_2_^*∗*^ = (9*a*_0_ − *a*_1_*a*_2_)/2(*a*_2_^2^ − 3*a*_1_). As $v_{2}^{*}=A=\left(-a_{2}+\sqrt{a_{2}^{2}-3a_{1}}\right)/3$, so $\left(9a_{0}-a_{1}a_{2}\right)/2(a_{2}^{2}-3a_{1})=\left(-a_{2}+\sqrt{a_{2}^{2}-3a_{1}}\right)/3$, which implies that

(47) $$\begin{array}{c} b=\frac{a^{2}d}{crN^{2}}\cdot \frac{-2a_{2}^{3}+9a_{1}a_{2}+2{\left(a_{2}^{2}-3a_{1}\right)}^{3/2}}{27}≕\bar{b}. \end{array}$$

Thus, when $\bar{b}<((cN+dA{)}^{2}/cdN^{2})\left((2a^{2}/r)A-a+aN\right)-ce/d+1≕{\bar{b}}_{1}$, *E*_3_ is locally unstable.

Lastly, when *f*(*v*) = 0 has two distinct positive real roots 0 < *v*_2_ < *v*_2_^*∗*^ = *A* < *v*_3_ < *u*^*∗*^, the variational matrices at *E*_4_ and *E*_5_ are the same as the variational matrix at *E*_3_ except that *A* is replaced by *v*_2_ and *v*_3_, respectively. We obtain the corresponding characteristic polynomials of those matrices which are the same as the characteristic polynomial of *L* except for replacing *A* by *v*_2_ and *v*_3_. By the same argument as above, when $\bar{b}<((cN+dv_{2,3}{)}^{2}/cdN^{2})\left((2a^{2}/r)v_{2,3}-a+aN\right)-ce/d+1≕{\bar{b}}_{2,3}$, *E*_4_ and *E*_5_ are locally unstable.

It is interesting to notice that our model system has 3 positive equilibria when the viral burst size is not too large and the relative immune killing rate falls into some intervals.  Proposition 8 gives conditions that ensure these equilibrium solutions are unstable. Biologically, when the relative immune killing rate and relative immune response decay rate fall into some ranges, we may genetically change the viral burst size to avoid coexistent equilibria.

### 2.4. Bifurcation Analysis

The dramatic changes of solutions may occur at bifurcations of parameter values. It is important to study bifurcation phenomena for any mathematical models. For our model (4), there are two types of bifurcations, transcritical bifurcations and Hopf bifurcations. For basic knowledge of Hopf bifurcations, we refer Hassard et al. [22].

A transcritical bifurcation occurs with *E*_1_ and *E*_2_. When *b* < *b*_*s*_1__, *E*_2_ is outside of the positive domain *D* and *E*_1_ is locally asymptotically stable. As *b* increases to *b*_*s*_1__ = 1 + *e*/*a*, *E*_2_ moves into *D* and it coalesces with *E*_1_ which is still locally asymptotically stable. But when *b*_*s*_1__ < *b* < *b*_*s*_2__ and *b* ∈ *I*_*p*_, the stability of these equilibrium points interchanges, which means that *E*_2_ is locally asymptotically stable while *E*_1_ is unstable. Notice that when *b* > *b*_0_ and *b* ∈ *I*_*n*_, *E*_2_ is locally unstable.

In order to study the Hopf bifurcation at *b* = *b*_0_, we look at the characteristic polynomial (38):

(48) $$\begin{array}{c} p\left(\;\lambda \;\right)=\left|\;\lambda I-L\;\right|=\left[\;\lambda -\left(\;m\bar{y}-n\;\right)\;\right]q\left(\;\lambda \;\right), \end{array}$$

where *q*(*λ*) = *λ*^3^ + *a*_1_*λ*^2^ + *a*_2_*λ* + *a*_3_. For convenience, we assume that (*b*_*s*_1__, *b*_0_) ⊂ *J*. From the derivation of Proposition 6, we know $m\bar{y}-n<0$. That is, *p*(*λ*) has a negative root $m\bar{y}-n<0$. Thus, the assumption (*b*_*s*_1__, *b*_0_) ⊂ *J* reduces the study of the quartic polynomial *p*(*λ*) to the cubic polynomial *q*(*λ*).

Consider each coefficient of *q*(*λ*) as a function of the parameter *b*. Then

(49) $$\begin{array}{c} q\left(\;\lambda \;\right)=\lambda^{3}+a_{1}\left(\;b\;\right)\lambda^{2}+a_{2}\left(\;b\;\right)\lambda +a_{3}\left(\;b\;\right), \end{array}$$

where *a*_1_(*b*) = (*re* + *ab* − *a* + *abe*)/*a*(*b* − 1), *a*_2_(*b*) = *re*(*be* + *b* − 1)/*a*(*b* − 1)^2^ + *re*(*ab* − *a* − *e*)(*r* − *a*)/*a*(*b* − 1)(*ab* − *a* + *re*), and *a*_3_(*b*) = *re*(*ab* − *a* − *e*)/*a*(*b* − 1).

The following theorem is our main result for occurring a Hopf bifurcation around *b*_0_, which appears in [16] as Theorem 3.12. For completion, we restate this theorem and related lemmas and corollary and give slight different proofs.

**Theorem 9 .**

There exists a neighborhood of *b*_0_, (*b*_0_ − *δ*_0_, *b*_0_ + *δ*_0_), such that for each *b* in this neighborhood *q*(*λ*) has a pair of complex conjugate eigenvalues *λ*(*b*) = *α*(*b*) ± *iβ*(*b*), where *α*(*b*) changes sign when *b* passes through *b*_0_ and *β*(*b*) > 0. Moreover, when *b* = *b*_0_, *α*(*b*_0_) = 0 and *α*′(*b*_0_) ≠ 0. Notice that *δ*_0_ can be made small enough so that (*b*_0_ − *δ*_0_, *b*_0_ + *δ*_0_)⊆*J*.

To prove this theorem, we need two lemmas about the properties of roots of cubic equations which appear in [16] as Lemmas 3.10 and 3.11.

**Lemma 10 .**

The cubic polynomial *λ*^3^ + *a*_1_*λ*^2^ + *a*_2_*λ* + *a*_3_ = 0 with real coefficients has a pair of pure imaginary roots if and only if *a*_2_ > 0 and *a*_3_ = *a*_1_*a*_2_. When it has pure imaginary roots, these imaginary roots are given by $\lambda =\pm i\sqrt{a_{2}}$, the real root is given by *λ* = −*a*_1_, and *a*_1_*a*_3_ > 0.

**Proof**

Suppose the cubic polynomial has a pair of complex roots *λ* = *u* ± *vi* and one real root *λ* = *λ*_0_. Then (*λ*^2^ − 2*uλ* + *u*^2^ + *v*^2^)(*λ* − *λ*_0_) = *λ*^3^ + *a*_1_*λ*^2^ + *a*_2_*λ* + *a*_3_. Expanding the left-hand side and then equating both sides give *a*_1_ = −(*λ*_0_ + 2*u*), *a*_2_ = *u*^2^ + *v*^2^ + 2*uλ*_0_, and *a*_3_ = −(*u*^2^ + *v*^2^)*λ*_0_. This implies that *λ*_0_ = −(*a*_1_ + 2*u*), *u*^2^ + *v*^2^ = *a*_3_/(*a*_1_ + 2*u*), and *a*_3_/(*a*_1_ + 2*u*) − 2*u*(*a*_1_ + 2*u*) = *a*_2_. The last equation yields 2(*a*_2_ + (*a*_1_ + 2*u*)^2^)*u* = *a*_3_ − *a*_1_*a*_2_. Thus, *u* = 0 if and only if *a*_2_ > 0 and *a*_3_ = *a*_1_*a*_2_. If *u* = 0, then *λ*_0_ = −*a*_1_ and *v*^2^ = *a*_2_, which follows that *v*^2^*a*_1_ = *a*_3_.

**Lemma 11 .**

Consider polynomial *λ*^3^ + *a*_1_(*τ*)*λ*^2^ + *a*_2_(*τ*)*λ* + *a*_3_(*τ*) = 0, where *a*_*k*_(*τ*) ∈ *C*^1^, *k* = 1,2, 3. Let *λ*(*τ*) = *α*(*τ*) + *iβ*(*τ*) be the roots of the polynomial. Suppose there is *τ*_0_ such that *α*(*τ*_0_) = 0 and *β*(*τ*_0_) ≠ 0. Moreover, if (*dα*/*dτ*)|_*τ*=*τ*_0__ = 0, then *a*_2_′(*τ*_0_)*a*_3_(*τ*_0_) = *a*_2_(*τ*_0_)[*a*_3_′(*τ*_0_) − *a*_2_(*τ*_0_)*a*_1_′(*τ*_0_)].

**Proof**

Differentiating the polynomial with respect to *τ* and evaluating the derivative at *τ*_0_, we notice that *α*(*τ*_0_) = *α*′(*τ*_0_) = 0, then we get, after equating the real part and the imaginary part, *β*′(*τ*_0_) = *a*_2_′(*τ*_0_)*β*(*τ*_0_)/(3*β*^2^(*τ*_0_) − *a*_2_(*τ*_0_)) = (*a*_3_′(*τ*_0_) − *β*^2^(*τ*_0_)*a*_1_′(*τ*_0_))/2*a*_1_(*τ*_0_)*β*(*τ*_0_). By Lemma 10, since *β*^2^(*τ*_0_) = *a*_2_(*τ*_0_) = *a*_3_(*τ*_0_)/*a*_1_(*τ*_0_), we obtain the desired result.

From the proofs of Lemmas 10 and 11, and Routh-Hurwitz Criterion, we have the following corollary about *q*(*λ*).

**Corollary 12 .**

There is a neighborhood of *b*_0_, (*b*_0_ − *δ*_2_, *b*_0_ + *δ*_2_), where *δ*_2_ < *b*_0_ − *b*_*s*_1__, such that *a*_2_(*b*) > 0 for all *b* ∈ (*b*_0_ − *δ*_2_, *b*_0_ + *δ*_2_), and the real part *α*(*b*) of the root *λ*(*b*) = *α*(*b*) + *iβ*(*b*) of *q*(*λ*) changes sign when *b* passes through *b*_0_.

**Proof**

Since *b* > 1 + *e*/*a*, *a*_1_(*b*) > 0 and *a*_3_(*b*) > 0. As *H*(*b*_0_) = *a*_1_(*b*_0_)*a*_2_(*b*_0_) − *a*_3_(*b*_0_) = 0, *a*_2_(*b*_0_) = *a*_3_(*b*_0_)/*a*_1_(*b*_0_) > 0. Since *a*_2_(*b*) is continuous with respect to *b*, there is a neighborhood of *b*_0_ such that *a*_2_(*b*) > 0 in that neighborhood. Its radius *δ*_2_ can be made small enough so that *δ*_2_ < *b*_0_ − *b*_*s*_1__ and *δ*_2_ < *δ*_1_. We know that *H*(*b*) is decreasing in this neighborhood (*b*_0_ − *δ*_2_, *b*_0_ + *δ*_2_). If *b* ∈ (*b*_0_ − *δ*_2_, *b*_0_), then *H*_2_ = *H*(*b*) > *H*(*b*_0_) = 0. Since *H*_1_ = *a*_1_(*b*) > 0 and *H*_3_ = *a*_3_(*b*)*H*(*b*) > 0, by Routh-Hurwitz Criterion *α*(*b*) < 0. If *b* ∈ (*b*_0_, *b*_0_ + *δ*_2_), then *H*_2_ = *H*(*b*) < *H*(*b*_0_) = 0. Since *a*_1_(*b*) > 0, *a*_2_(*b*) > 0, and *a*_3_(*b*) > 0, from the proof of Lemma 10 we have *α*(*b*) = −*H*(*b*)/2(*a*_2_(*b*)+(*a*_1_(*b*) + 2*α*(*b*))^2^) > 0 and *α*(*b*_0_) = 0. Thus the sign of *α*(*b*) changes when *b* passes through *b*_0_.

We now can prove our main Theorem 9.

**Proof**

By Property 7 in previous section, *b*_0_ > 1 + *e*/*a* and *H*(*b*_0_) = 0. Then, for *b* > 1 + *e*/*a*, *a*_1_(*b*) > 0 and *a*_3_(*b*) > 0; hence *a*_2_(*b*_0_) = *a*_3_(*b*_0_)/*a*_1_(*b*_0_) > 0. By Lemma 10, *p*(*λ*) has a pair of purely imaginary roots, $\lambda (b_{0})=\pm i\beta (b_{0})=\pm i\sqrt{a_{2}(b_{0})}$, and the real root is −*a*_1_(*b*_0_) < 0. Since *β*(*b*_0_) > 0 and *β*(*b*) is continuous, we can find a neighborhood of *b*_0_ so that *β*(*b*) > 0 in this neighborhood and its radius can be chosen so that *δ*_0_ < min⁡{*δ*_1_, *δ*_2_} and (*b*_0_ − *δ*_0_, *b*_0_ + *δ*_0_)⊆*J*. By the above corollary, in the interval (*b*_0_ − *δ*_0_, *b*_0_ + *δ*_0_), *α*(*b*) changes sign when *b* passes through *b*_0_. Finally, we claim that *α*′(*b*_0_) ≠ 0. By way of contradiction, if *α*′(*b*_0_) = 0, then by Lemma 11 we have *a*_2_′(*b*_0_)*a*_3_(*b*_0_) = *a*_2_(*b*_0_)(*a*_3_′(*b*_0_) − *a*_1_′(*b*_0_)*a*_2_(*b*_0_)). Then this yields *H*′(*b*_0_) = *H*(*b*_0_)*a*_2_′(*b*_0_)/*a*_2_(*b*_0_) = 0, a contradiction. This completes the proof.

Combining Proposition 6 and applying this theorem, we can obtain a statement about Hopf bifurcations of our model.

**Theorem 13 .**

Assuming (*b*_*s*_1__, *b*_0_) ⊂ *J*, for system (4) $\dot{X}=f(X,b)\;\;f(E_{2},b)=0$ for all *b* > *b*_*s*_1__. The variational matrix of *f* at *E*_2_, *L* = (∂*f*/∂*X*)(*E*_2_, *b*), has 2 strictly negative roots and 2 conjugate complex roots *λ*(*b*) = *α*(*b*) ± *iβ*(*b*) in the neighborhood (*b*_0_ − *δ*_0_, *b*_0_ + *δ*_0_) of *b*_0_, in which *β*(*b*) > 0, *α*(*b*) changes sign when *b* passes through *b*_0_, and *α*′(*b*_0_) ≠ 0. Consequently, in a neighborhood *U* of *E*_2_ and for any *b* ∈ (*b*_0_ − *δ*_0_, *b*_0_ + *δ*_0_), the system $\dot{X}=f(X,b)$ has nontrivial periodical solutions in *U*.

Because we cannot find explicit algebraic expression for *b*_0_, it is very hard to study the nature of periodical solutions that occur around *E*_2_ when *b* is close to *b*_0_ such as the amplitudes, periods, and their stability. However, we can make some statements about the general properties of these periodical solutions as follows.

- If *E*_2_ is stable but not asymptotically stable, then any solution of system (3) in *U* is periodical in a surface.
- If *E*_2_ is asymptotically stable, then there is an asymptotically stable periodical solution $\bar{X}(t)$ in *U* when *b* is close to *b*_0_. Any solution inside $\bar{X}$ will spiral into *E*_2_ when time is increasing and any solution in *U* outside $\bar{X}$ will spiral and emerge into $\bar{X}$ when time is increasing.
- If *E*_2_ is unstable, then there is an asymptotically stable periodical solution $\tilde{X}(t)$ in *U* when *b* is close to *b*_0_. Any solution starting at nearby *E*_2_ will spiral out and emerge into $\tilde{X}$ when time is increasing, and any solution in *U* nearby outside $\tilde{X}$ will move away from $\tilde{X}$ when time is increasing.

We will use numerical simulations to confirm some of these situations. Lastly, we will not conduct the analysis for the bifurcations arising around the positive equilibrium points *E*_4_ and *E*_5_ because their formulas are extremely cumbersome and therefore we will treat this by numerical simulations in the next section.

We close Section 2 with the following theorem that summarizes the main results about the our model and its biological implications.

**Theorem 14 .**

The dynamical behaviors of system (4) can be described as follows.

- When (*r*/*a*)(1/*N* − 1) < *K* < 1/(*a* + *e* − *aN*), $N<1+(1/2)\left(e/a+1/r-\sqrt{(e/a+1/r{)}^{2}+4/r}\right)$, and $b>\bar{b}$ with *b* > *b*_*s*_1__, system (4) has at most 3 equilibrium solutions: *E*_0_, *E*_1_, and *E*_2_. *E*_0_ is unstable. *E*_1_ is globally asymptotically stable if *b* < *b*_*s*_1__ and *N* > 1 and unstable if *b* > *b*_*s*_1__. *E*_2_ is locally asymptotically stable if *b* ∈ (*b*_*s*_1__, *b*_*s*_2__)∩*J*.
- When either *K* ≤ (*r*/*a*)(1/*N* − 1) or *K* > 1/(*a* + *e* − *aN*) and $b=\bar{b}$ with *g*(*K*) > 0, system (4) has a unique positive equilibrium solution: *E*_3_. *E*_3_ is unstable if $\bar{b}<{\bar{b}}_{1}$.
- When either *K* ≤ (*r*/*a*)(1/*N* − 1) or *K* > 1/(*a* + *e* − *aN*) and $b<\bar{b}$ with *g*(*K*) > 0, system (4) has two distinct positive equilibrium solutions: *E*_4_ and *E*_5_. *E*_4_ and *E*_5_ are unstable if $\bar{b}<{\bar{b}}_{2,3}$.
- When (*b*_*s*_1__, *b*_0_) ⊂ *J*, there exist a neighborhood (*b*_0_ − *δ*_0_, *b*_0_ + *δ*_0_) of *b*_0_ and a neighborhood *U* of *E*_2_, such that for any *b* ∈ (*b*_0_ − *δ*_0_, *b*_0_ + *δ*_0_), system (4) has nontrivial periodical solutions in *U*.

Biologically, we have interpreted most parts of this theorem. We may emphasize some biological implications here. If the burst size is smaller than the critical value *b*_*s*_1__ and the relative immune decay rate is greater than 1, the virotherapy always fails. If the burst size is greater than *b*_*s*_1__ and smaller than another critical value *b*_*s*_2__, the immune free equilibrium is stable; that is, the tumor cells, infected tumor cells, and viruses coexist. When the relative immune killing rate is too small or too big compared to a relative immune survival rate (1/*N*), according to the burst size, the model can have one more or two more positive equilibria, and these positive equilibria are unstable. This gives a chance for the model to have periodical solutions. That is, the virus cannot eradicate the tumor and the virus, tumor cells, and immune cells fight each other forever.

For positive equilibria, *E*_3_, *E*_4_, and *E*_5_, *E*_3_ is difficult to obtain in practice because it requires a particular threshold of the burst size. In rat experiments, the virus burst size can be genetically changed as we want, but usually, we can ensure a range of the burst size in the process of genetic modification. *E*_4_ and *E*_5_ are unstable if the burst size is smaller than a threshold. Biologically, these two equilibria are not important because of their instability. The immune free equilibrium *E*_2_ can be stable. If the burst size is made big enough, the tumor cell portion will be small in *E*_2_. On the other hand, the model can have periodic solutions. This may provide an opportunity for combining surgery with the phase where the tumor cell portion is in the lowest state.

## 3. Numerical Study and Discussion

### 3.1. Numerical Study

In order to demonstrate our analytical results about dynamical behaviors of the model, we use some data of parameter values from our previous research [14] to conduct numerical computations for all dynamical characteristics and perform some numerical simulations by using Matlab. The data of parameter values we used from [14] is recorded in Table 1. We applied the algorithm of the Newton method for finding Hopf bifurcation points [23], and Matlab codes are available upon request.

After nondimensionalization, the parameter values are *r* = 0.36, *a* = 0.11, *c* = 0.48, *d* = 0.16, *e* = 0.2, *m* = 0.6, and *n* = 0.036. Then *b*_*s*_1__ = 1 + *e*/*a* = 2.82. Solving *H*(*b*) = 0 gives 2 conjugate complex roots *b* = 0.8353 ± 0.2312*i*, and 2 real roots *b* = 0.299 and *b* = 19.012. Therefore, *I*_0_ = {19.012}, *I*_*p*_ = (2.82,19.012), *I*_*n*_ = (19.012, +*∞*), and hence *b*_*s*_2__ = *b*_0_ = 19.012. On the other hand, all coefficients of the cubic equation are *a*_2_ = −2.8964, *a*_1_ = 0.1603, and $\bar{b}=7.4455$. Considering the case with the burst size *b* = 9, we find all equilibrium solutions of system (4):

(50) E0=0,0,0,0,E1=1,0,0,0,E2=0.2273,0.0584,2.3377,0,E4=0.48328,0.06,1.49471,0.67571,E5=0.24994,0.06,2.25837,0.07261.

By the analysis of previous section, *E*_0_ is always unstable. The equilibrium point *E*_1_ is locally asymptotically stable if *b* < 2.82 and unstable if *b* > 2.82. For the equilibrium point *E*_2_, since $\bar{y}=0.0584<N=n/m=0.06$ and *b* = 9 ∈ (*b*_*s*_1__, *b*_*s*_2__) = (2.82,19.012), it is locally asymptotically stable. In order to check the stability of the equilibrium point *E*_4_, we need to compute the Jacobian matrix of *F* at this point, which is *L* = (∂*F*/∂*X*)(*E*_4_). Using the Matlab, we can calculate 4 eigenvalues of *L*, which are −1.69849, −0.00803, and −0.07654 ± 0.20995*i*. This guarantees the locally asymptotical stability of *E*_4_. For the last equilibrium point *E*_5_, we know that *v*_3_ = 2.258366, which is the largest root of *f*(*v*) = 0. Then, we can compute the quantity ((*cN* + *dv*_3_)^2^/*cdN*^2^)((2*a*^2^/*r*)*v*_3_ − *a* + *aN*) − *ce*/*d* + 1 = 27.052. As $\bar{b}<27.052$, *E*_5_ is unstable.

When *b* = 20, the bifurcation analysis gives us the appearance of periodical solutions around the equilibrium point *E*_2_ = (0.09569,0.03011,2.86098,0). However, in this case, two positive equilibrium points *E*_4_ and *E*_5_ do not exist. Now we fix the burst size *b* = 20 and other parameters, considering *m* as a variable parameter. Observe that when *m* = 1.17, we have two positive equilibrium points: *E*_4_ and *E*_5_. The eigenvalues of the Jacobian matrix at *E*_5_ are −1.26885, 0.00075, and −0.00003 ± 0.23144*i*, whereas when *m* = 1.18, we also have two positive equilibrium points: *E*_4_ and *E*_5_. The eigenvalues at *E*_5_ are 1.26023, 0.00045, and 0.000455 ± 0.23025*i*. This partially shows the existence of the Hopf bifurcation point 1.17 < *m*_0_ < 1.18. Using the Newton method for the computation of Hopf bifurcation points, we find *m*_0_ = 1.1706885699.

For the sake of demonstration and simplicity, we conduct numerical simulations based on nondimensionalized model. Therefore, the unit of the tumor cells, infected tumor cells, viruses, and innate immune cells are not absolute numbers and are only relative numbers. For example, the quantity of tumor cells in all figures is the portion of tumor cells over the tumor carrying capacity. Similarly, other quantities have the same meaning. We just indicate them as relative tumor cells and so on in the figures. For the time, it can be considered as runs of infected tumor bursting since *τ* = *δt*, or simply, relative time.

**Figure 1:**
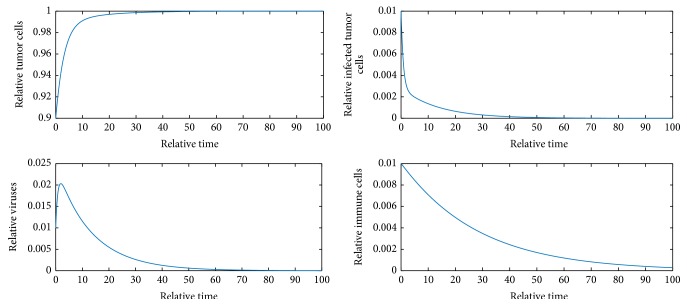
Dynamics of the model when *b* = 2 and initial values are *x* = 0.9, *y* = 0.01, *v* = 0.01, and *z* = 0.01.

Figure 1 shows that *E*_1_ is locally asymptotically stable.

**Figure 2:**
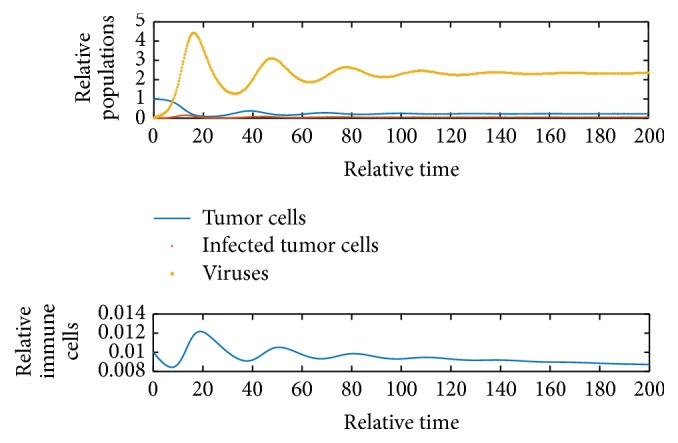
Dynamics of the system when *b* = 9 and initial values are *x* = 0.99, *y* = 0.01, *v* = 0.01, and *z* = 0.01.

**Figure 3:**
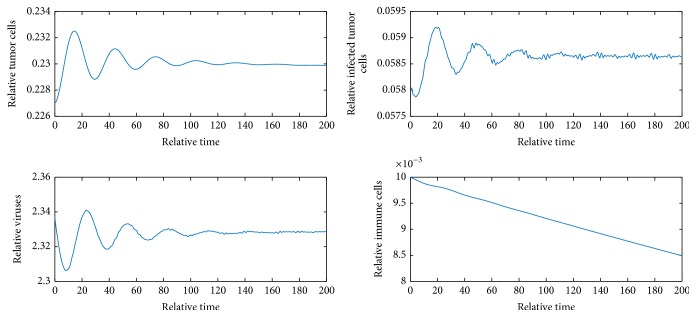
Dynamics of the system when *b* = 9 and initial values are *x* = 0.227, *y* = 0.058, *v* = 2.337, and *z* = 0.01.

**Figure 4:**
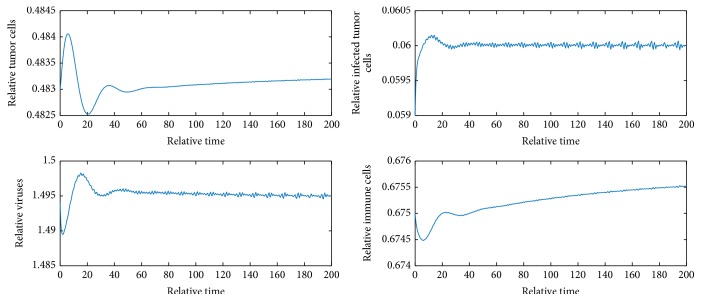
Dynamics of the system when *b* = 9 and initial values are *x* = 0.483, *y* = 0.059, *v* = 1.494, and *z* = 0.675.

**Figure 5:**
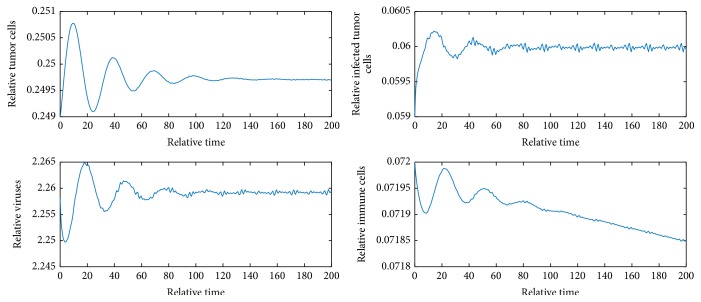
Dynamics of the system when *b* = 9 and initial values are *x* = 0.249, *y* = 0.059, *v* = 2.258, and *z* = 0.072.

Figure 2 shows *E*_1_ is locally unstable since *b* = 9 > 1 + *e*/*a* = 2.82.  Figure 3 shows *E*_2_ is locally asymptotically stable because *b* is between *b*_*s*_1__ and *b*_*s*_2__ and *y* = 0.058 < *N* = 0.06.  Figure 4 shows *E*_4_ is locally asymptotically stable since all eigenvalues of the variational matrix at *E*_4_ are negative.  Figure 5 shows *E*_5_ is locally unstable sine $\bar{b}<{\bar{b}}_{3}$.

**Figure 6:**
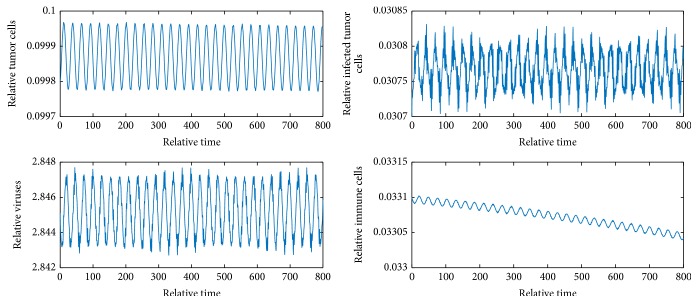
Periodic solutions from Hopf bifurcation when *m* = 1.17. The initial values are *x* = 0.0998, *y* = 0.0307, *v* = 2.8451, and *z* = 0.0331.

**Figure 7:**
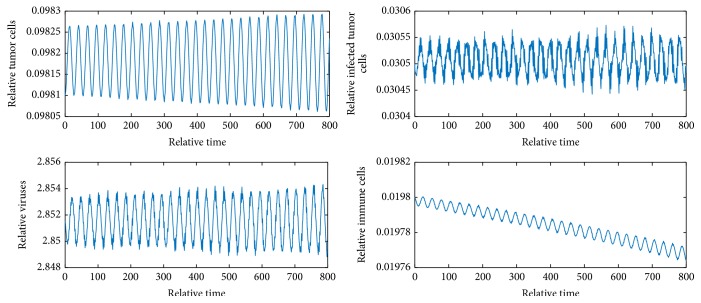
Periodic solutions from Hopf bifurcation when *m* = 1.18. The initial values are *x* = 0.0981, *y* = 0.0305, *v* = 2.8515, and *z* = 0.0198.

**Figure 8:**
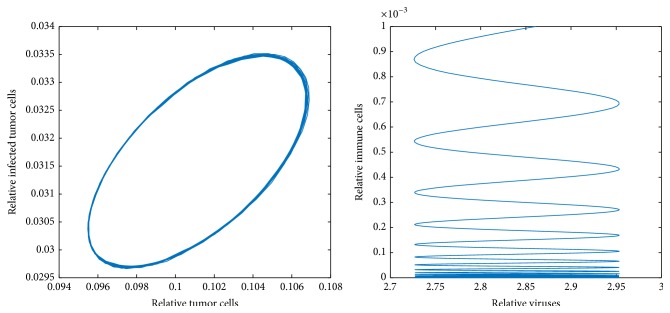
Periodic solutions from Hopf bifurcation.

Figures 6–8 show periodic solutions rising from Hopf bifurcations.

**Figure 9:**
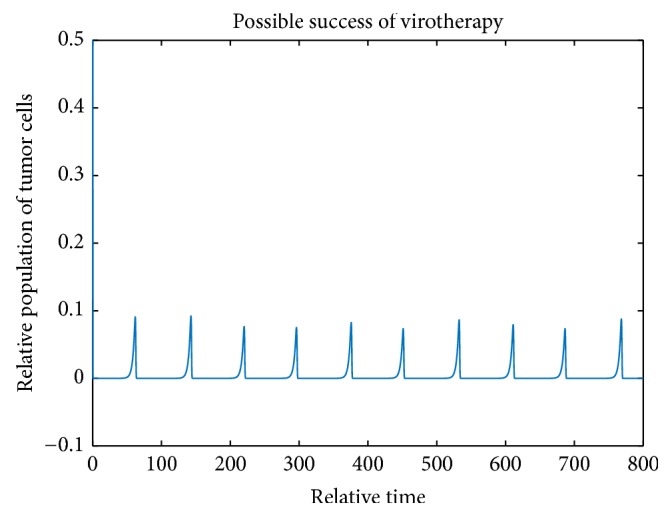
The tumor cell population when *b* = 1000. The initial values are *x* = 0.5, *y* = 0.5, *v* = 1.5, and *z* = 1.

Figure 9 shows the tumor cell population when the burst size *b* is 1000.

### 3.2. Discussion

The dynamics of oncolytic virotherapy without the presence of the innate immune response is largely determined by the oncolytic viral burst size as studied by Tian [16]. Specifically, there are two threshold values of the burst size: below the first threshold, the tumor always grows to its maximum (carrying capacity) size; while passing this threshold, there is a locally stable positive equilibrium solution appearing through transcritical bifurcation; while at or above the second threshold, there exits one or three families of periodic solutions arising from Hopf bifurcations. And it also suggests that the tumor load can drop to an undetectable level either during the oscillation or when the burst size is large enough. When the model for oncolytic virotherapy is with the presence of the innate immune response, the dynamics becomes more complicated. There are several critical values for the oncolytic viral burst size *b*, for example, *b*_*s*_1__, *b*_*s*_2__, $\bar{b}$, and *b*_0_, where $\bar{b}$ is a function of the relative innate immune response killing rate *K* and relative innate immune decay rate *N*, which we combine with innate immune parameters *K* and *N* to describe the dynamics of our model (4). When *b* is smaller than $\bar{b}$ and *K* and *N* satisfy some constraints, system (4) has 5 equilibrium solutions and 2 of them are positive, while these two positive equilibrium points coalesce when $b=\bar{b}$ and there are some constraints for the relative innate immune killing rate *K* and relative innate immune decay rate *N*. When *b* is greater than *b*_*s*_1__ or $\bar{b}$ and *K* and *N* fulfill the complement conditions, system (4) has at most 3 equilibrium solutions with 0 innate immune components. An interesting fact is that the equilibrium points where Hopf bifurcations arise for both models (4) and in [16] are corresponding to each other. Therefore, we may conclude that the innate immune response complicates the oncolytic virotherapy in the way of creating more equilibrium solutions when the oncolytic viral burst size is not too big, say less than $\bar{b}$, while the dynamics is similar to the system without the presence of the innate immune response when the oncolytic viral burst size is big.

As we mention in the Introduction, the major challenge in current medical practice of oncolytic viral therapy is the complexity of the immune responses [4]. The innate immune response tends to reduce the efficacy of oncolytic viral treatments by reducing new virus multiplication and blocking the spreading of infection. However, the stimulated adaptive immune response tends to reduce tumor cells. These two opposite functions increase the complexity of oncolytic viral therapy. A balance between two functions needs to be determined in order to improve the efficacy of oncolytic virotherapy. This is a very subtle question. There are not too much experimental or clinical data about this balance in the literature. Therefore, a mathematical study of this question is highly demanded. Our current mathematical model is only dealing with the innate immune system. The extension of our model to incorporate the adaptive immune system is expected. We plan to carry out such study in other articles.

## Figures and Tables

**Table 1:** Parameters and their values.

Parameters	Description	Values	Dimensions
*λ*	Tumor growth rate	2 × 10^−2^	1/h
*θ*	Density of tumor cells	10^6^	cells/mm^3^
*β*	Infection rate of the virus	7/10 × 10^−9^	mm^3^/h virus
*μ*	Immune killing rate of infected tumor cells	2 × 10^−8^	mm^3^/h immune cell
*δ*	Death rate of infected tumor cells	1/18	1/h
*b*	Burst size of free virus	50	viruses/cell
*k*	Immune killing rate of virus	10^−8^	mm^3^/h immune cell
*γ*	Clearance rate of the virus	2.5 × 10^−2^	1/h
*s*	Stimulation rate of the virus by infected cells	5.6 × 10^−7^	mm^3^/h infected cell
*ρ*	Immune clearance rate	20 × 10^−8^	mm^3^/h cell
